# Antidotal and protective effects of mangosteen (*Garcinia mangostana*) against natural and chemical toxicities: A review

**DOI:** 10.22038/IJBMS.2023.66900.14674

**Published:** 2023

**Authors:** Aidin Mohammadi Zonouz, Mahboobeh Ghasemzadeh Rahbardar, Hossein Hosseinzadeh

**Affiliations:** 1 School of Pharmacy, Mashhad University of Medical Sciences, Mashhad, Iran; 2 Pharmaceutical Research Center, Pharmaceutical Technology Institute, Mashhad University of Medical Sciences, Mashhad, Iran; 3 Department of Pharmacodynamics and Toxicology, School of Pharmacy, Mashhad University of Medical Sciences, Mashhad, Iran

**Keywords:** Analgesics, Anti-inflammatory agents, Anti-oxidants, Apoptosis, Hypoglycemic agents, Neuroprotective agents, Phytotherapy, Xanthones

## Abstract

Chemical and natural toxic compounds can harm human health through a variety of mechanisms. Nowadays, herbal therapy is widely accepted as a safe method of treating toxicity. *Garcinia mangostana* (mangosteen) is a tree in the Clusiaceae family, and isoprenylated xanthones, its main constituents, are a class of secondary metabolites having a variety of biological properties, such as anti-inflammatory, anti-oxidant, pro-apoptotic, anti-proliferative, antinociceptive, neuroprotective, hypoglycemic, and anti-obesity. In this review, the protective activities of mangosteen and its major components against natural and chemical toxicities in both *in vivo* and *in vitro* experiments were evaluated. The protective effects of mangosteen and its components are mediated primarily through oxidative stress inhibition, a decrease in the number of inflammatory cells such as lymphocytes, neutrophils, and eosinophils, reduction of inflammatory mediators such as tumor necrosis factor-alpha (TNF-α), interleukin-1 (IL-1), interleukin-6 (IL-6), interleukin-8 (IL-8), cyclooxygenase-2 (COX-2), prostaglandin (PG) E2, inducible nitric oxide synthase, and nuclear factor-ĸB (NF-ĸB), modulation of apoptosis and mitogen-activated protein kinase (MAPK) signaling pathways, reducing p65 entrance into the nucleus, α-smooth muscle actin (α-SMA), transforming growth factor β1 (TGFβ1), improving histological conditions, and inhibition in acetylcholinesterase activity.

## Introduction

Humans and animals are now exposed to different types of toxic substances, either directly or indirectly, through a variety of routes, including food, water, soil, and air (1). Toxic agents can cause different disorders such as nephrotoxicity (2), neurotoxicity (3), hepatotoxicity (4), and cardiotoxicity (5) in human or animal bodies. The most frequent mechanism regulating chemical and natural toxicities is oxidative stress. The abundance of nucleobase products characteristic of the oxygen assault on deoxyribonucleic acid (DNA) in cultured cells and animals exposed to carcinogenic agents provides the most compelling evidence that toxicants cause genotoxic damage via an oxidative process. The potential of toxicants to generate reactive radicals, which cause DNA damage, lipid peroxidation, and protein sulfhydryl depletion, has been demonstrated (6, 7). Different toxins can cause inflammation, and these events are likely to involve a variety of pathways. An increase in the formation of reactive oxygen species (ROS) and redox-related alterations are frequently strongly linked to inflammatory processes. Likely, the first event leading to inflammation in the case of some kinds of toxicants is their capacity to stimulate ROS generation (8). Toxins are also well known for causing apoptotic cell death and playing a role in a variety of clinical diseases. The regulation of toxicant-induced apoptotic pathways appears to be largely dependent on oxidative stress (9). In apoptosis, several gene families are involved or work together, including the caspases, inhibitors of apoptosis proteins, the B cell lymphoma-2 family of genes, the tumor necrosis factor (TNF) receptor gene superfamily, and the p53 gene (10).

Many chemical medications, such as cimetidine, cilastatin, dexrazoxane, and β-blockers, are used to treat these disorders (11, 12), and despite their protective action against toxic agents, they have numerous side effects, including fatigue, hypotension, dizziness, diarrhea, and impotence (13). As a result, seeking out new medications to combat the harmful consequences of toxins is essential.

Herbs and spices have been employed to enhance the flavor of meals and beverages around the world for centuries, as well as being used as protective agents. Today, researchers are focusing more on the possible benefits of herbal drugs as alternative therapies for illness prevention or as antitoxic agents, due to their potential efficacy, low toxicity, and minimal side effects (14-18). 

Mangosteen,* Garcinia mangostana* Linn., a member of the Clusiaceae family, is a tropical evergreen tree that is native to Southeast Asia with a height of 6-25 m and a dark-brown or practically black bark. Its product, mangosteen, is a reddish/dark purple fruit that is regarded as “the queen of fruits” and has a juicy, soft, edible pulp, and delicious flavor. In folk medicine, mangosteen pericarp has been used to treat fever, convulsions, diarrhea, dysentery, stomach discomfort, trauma, pain, infected wounds, suppuration, and chronic ulcers (19, 20). 

**Figure 1:**
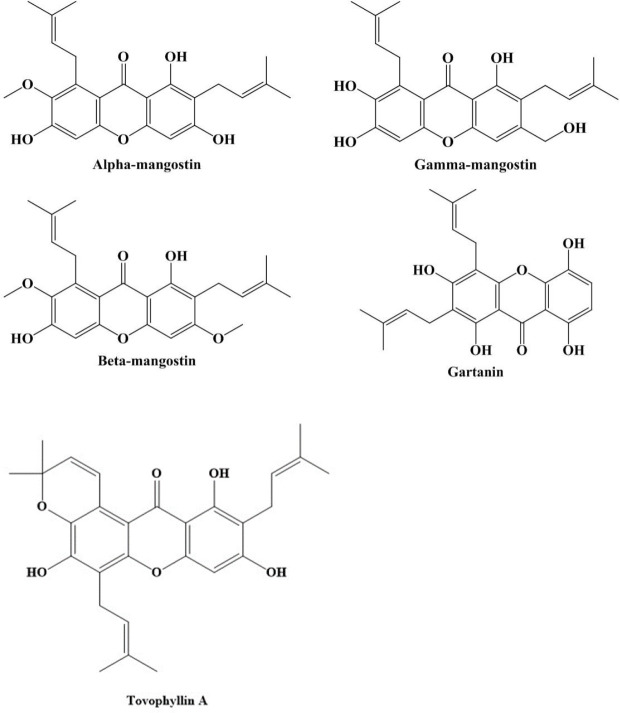
Structures of effective compounds of mangosteen

**Figure 2:**
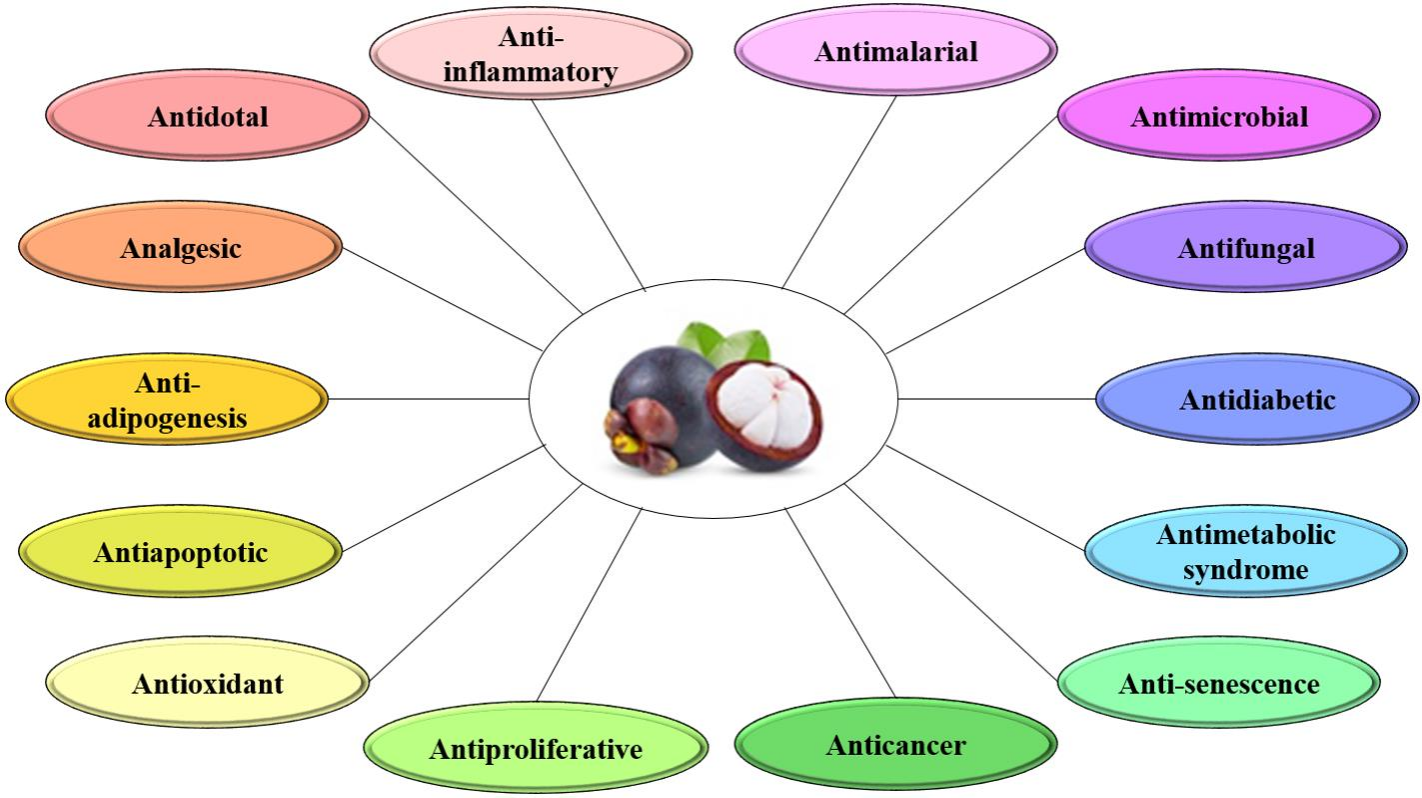
Pharmacological effects of mangosteen and its components

Mangosteen contains phenolic acids, xanthones, prenylated benzophenone derivatives, flavonoids, anthocyanins, and condensed tannins. Furthermore, it has been hypothesized that the pericarp of the mangosteen is a rich source of oligomeric proanthocyanidins with B-type linkages. Alpha-mangostin (α-MG) and gamma-mangostin (γ-MG) are the most common xanthones in mangosteen fruits, although there are also beta-mangostin (β-MG), gartanin, and other xanthones in mangosteen (Figure 1). In addition, according to previous research, most of the biological activities of *G. mangostana* are linked to the quantity of α-MG (21, 22). Mangosteen and its components have been shown to have medical and pharmacological characteristics, including antimalarial (23), anti-metabolic syndrome (19), antimicrobial (24), antifungal (25), antidiabetic (26), anticancer (27), antiproliferative (28), anti-adipogenesis (29), anti-oxidant, anti-apoptotic, anti-inflammatory, analgesic (30), and antidotal (31) activities (Figure 2). 

Since mangosteen and its main constituents are potent anti-oxidants, anti-inflammatory, and anti-apoptotic agents and can regulate different cellular pathways, including the mitogen-activated protein kinase (MAPK) signaling pathway, they might be effective in managing natural and chemical toxicities. Hence, the protective effects of mangosteen and its main constituents, essential oils, and extracts against various natural and chemical toxic compounds have been reviewed in this article to help expand the mangosteen and its constituents’ application as protective agents and potential lead compounds against toxicities. 

***Methods***

In this comprehensive review article, our team argued various documents in Google Scholar, PubMed, Web of Science, and Scopus. This publication contains both *in vitro* and *in vivo *studies. This review did not consider any time constraints. The keywords for this study were *Garcinia mangostana*, mangosteen, xanthones, α-mangostin, natural toxins, chemical toxicity, nephrotoxins, neurotoxins, hepatotoxins, and cardiotoxins. The protective effects of mangosteen were investigated under two headings: biological and chemical toxic substances.

***Mangosteen and its main constituents against biological agents-induced toxicity***

**Figure 3:**
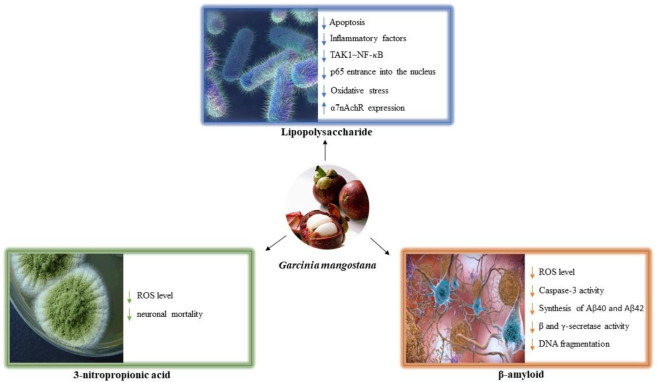
The mechanism of protective effects of mangosteen on natural toxicant-induced changes

This section discusses the antidotal and protective actions of mangosteen and its main constituents against several biological agents that cause toxicity. In addition, in this part, certain key defensive mechanisms are discussed (Figure 3).

***Lipopolysaccharide***

Lipopolysaccharide (LPS), a glycolipid generated by most gram-negative bacteria, is one of the most researched bacterial surface compounds. LPS is an amphiphilic molecule made up of three distinct regions, which are lipid A, the core region, and the O-antigen polysaccharide (32). The toxic effects of LPS in mammalian cells are caused by the lipid A portion binding to toll-like receptors (TLRs), which stimulate the innate immune system and trigger the production of inflammatory cytokines such as tumor necrosis factor-α (TNF-α), interleukin-1 (IL-1), and interleukin-6 (IL-6), which lead to a potentially deadly systemic inflammatory response known as “septic shock” (32, 33). Below, we discuss intestinal inflammation, lung injury, the cytotoxicity of human gingival fibroblasts, and liver failure. 

***Intestinal inflammation***

In a study, the anti-inflammatory effect of α-MG was assessed by administering α-MG (2.5, 5, and 10 µM, for 1 h) to an LPS-induced inflammation model of rat intestinal epithelial cells (IEC-6 cells) and the outcomes showed that α-MG administration demonstrated anti-inflammatory and anti-apoptotic properties by reducing apoptosis, inflammatory factors (nitric oxide (NO), prostaglandin (PG) E2, IL-6, TNF-α, and IL-1β) production, activation of transforming growth factor-activated kinase 1-nuclear factor-κB (TAK1–NF-κB) signaling pathway-related proteins, and p65 [involved in nuclear translocation and activation, nuclear factor- kappa-B (NF-κB) heterodimer formation] entrance into the nucleus in LPS-stimulated IEC-6 cells (34).

**Figure 4:**
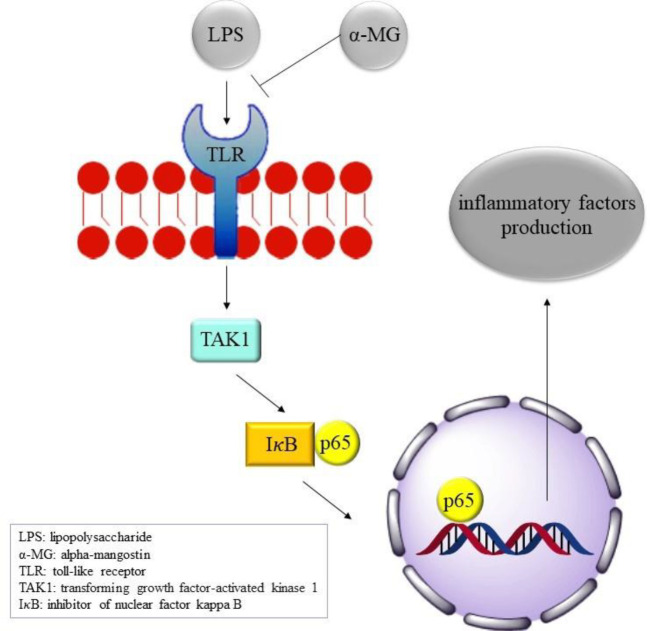
α-mangostin antidotal molecular mechanism against LPS-induced intestinal inflammation

As a consequence, α-MG administration has an anti-inflammatory effect on LPS-induced intestinal inflammation by reducing apoptosis, inflammatory factors (NO, PGE2, IL-6, TNF-α, and IL-1β production, activation of TAK1–NF-𝜅B signaling pathway-related proteins, and p65 entrance into the nucleus (Figure 4).

***Lung injury***

Yang *et al*. examined the role of α-MG in the cholinergic anti-inflammatory pathway (CAP) and its therapeutic potential in the treatment of acute lung injury (ALI). They administered α-MG (40 mg/kg, 3 days, PO) to male Sprague Dawley rats before inducing ALI with an injection of LPS. Also, for the *in vitro* tests, they employed RAW264.7 cells to confirm the effects of α-MG ((5 μg/ml), at various times (0.5, 1, 2, 4, and 6 h)) on CAP. The findings revealed that α-MG reversed the decline in α7 nicotinic acetylcholine receptor (α7nAchR) expression in the lungs of ALI rats and enhanced α7nAchR and choline acetyltransferase (ChAT) expression in RAW 264.7 cells. Also, α-MG affected acetylcholinesterase (AChE) expression at 5 g/ml and its catalytic activity was lowered by almost 95%. Altogether, α-MG injection resulted in NF-κB suppression and acute inflammatory remission (35).

In another study, researchers evaluated the therapeutic effects of α-MG (15 and 45 mg/kg/day, 3 days, PO) on LPS induce ALI in male Sprague Dawley rats. In ALI rats, it was discovered that α-MG therapy improved histological conditions (reduced interalveolar septal thickening, alveolar hemorrhage, and cells infiltration), lowered leucocyte counts, declined oxidative stress (a little recovery in superoxide dismutase (SOD) activity and reversed the elevation of malondialdehyde (MDA)), and decreased TNF-α levels. Also, α-MG therapy reduced the expressions of nicotinamide phosphoribosyltransferase (NAMPT) and sirtuin 1 (Sirt1(, which was accompanied by a synchronized drop in nicotinamide adenine dinucleotide (NAD) and TNF-α. α-MG also inhibited high mobility group box 1 (HMGB1), TLR4, and p-p65 in RAW 264.7 cells. These findings implied that α-MG treatments reduced NAMPT/NAD levels, which helped to relieve TLR4/NF-κB-mediated inflammation in macrophages, which is critical for ameliorating ALI in rats (36). 

As a result, α-MG treatment protects against LPS-induced lung damage through mechanisms such as increased peripheral acetylcholine and α7nAchR expression, as well as inhibition of TLR4/NF-κB mediated inflammatory responses via modification of NAMPT/NAD.

***Human gingival fibroblasts cytotoxicity***

Human gingival fibroblasts were exposed to *Porphyromonas* *gingivalis* LPS and then treated with various concentrations of α-MG (0, 0.5, 1, 1.5, and 2 µg/ml, 24 hr). The results from the investigation showed that α-MG attenuates the expression of IL-6 and interleukin-8 (IL-8) in *P. gingivalis* LPS-treated human gingival fibroblasts (37).

Therefore, α-MG inhibits the production of inflammatory cytokines IL-6 and IL-8 in *P. gingivalis *LPS-treated human gingival fibroblasts.

***Liver failure***

The hepatoprotective effect of α-MG (12.5, 25 mg/kg/day, 7 days, i.g.) on LPS/D-galactosamine-induced acute liver failure was studied and the findings revealed that α-MG protects the liver against the pathogenic effects of LPS/D-galactosamine by reducing hepatic MDA levels, serum alanine aminotransferase (ALT), aspartate transaminase (AST), TNF-α, IL-1β, IL-6 levels, and recovering hepatic glutathione (GSH), SOD, and catalase (CAT) activities. They also discovered that α-MG suppressed LPS/D-galactosamine-induced TLR4 expression and NF-κB activation while upregulating nuclear-related factor 2 (Nrf2) and heme oxygenase-1 expression (HO-1) (38).

Overall, α-MG protected against LPS/D-galactosamine-induced acute liver failure via activating Nrf2 and blocking the TLR4 signaling pathway. Taken together, α-MG might be a promising treatment for LPS/D-galactosamine-induced acute liver failure.

Table 1 also includes information on inflammation of the nervous system and murine macrophages.

***β*** ***-*** ***Amyloid***

Healthy neural and non-neural cells, such as skin and gut, release β-amyloid (Aβ), which circulates in both human cerebrospinal fluid and blood. Normally, low-density lipoproteins (LDL) receptor-related protein 1 transports β-amyloids through the blood-brain barrier. The clearance process by LDL receptor-related protein 1 is disturbed in Alzheimer’s brains, causing the peptide to accumulate and aggregate. Vascular endothelial cells, astrocytes, or oligodendrocytes may all experience mitochondrial malfunction and degeneration as a result of β-amyloid harmful effects. In Alzheimer’s disease, mitochondrial dysfunctions are among the neurotoxic processes linked to β-amyloid (39). In an *in vivo *study on mice and *Caenorhabditis elegans*, Aβ maintains a low concentration and has a normal physiological function (40). Typically, LDL receptor-related protein 1 (LRP1) transports Aβ across the blood-brain barrier; however, factors like aging, oxidative stress, and gene mutation impair LRP1 clearance, resulting in peptide buildup and aggregation, which play a crucial role in the pathogenesis of Alzheimer’s disease by causing neurotoxicity and cell death mostly through the generation of ROS (39, 41).

α-MG (0.5, 5, and 50 nM, 24 hr) attenuated the neurotoxicity caused by Aβ-(1-40) or Aβ-(1-42) oligomers (EC_50_=3.89 nM and 4.14 nM, respectively) in cerebral cortex neurons of day 17 rat embryos, as determined by decreased cell viability and impaired neurite outgrowth. α-MG can also bind to Aβ, stabilizing the α-helical conformation. Furthermore, it can directly dissociate Aβ-(1-40) and Aβ-(1-42) oligomers and disrupt pre-formed fibrils while blocking fibril formation (42). It was demonstrated that preincubating SK-N-SH cells with mangosteen extract (50-400 μg/ml) 30 min before exposure to Aβ-(1-42) could prevent the unpleasant effects of Aβ-(1-42) including cytotoxicity, increased intracellular ROS levels, caspase-3 activity, and changed the cellular proteome (43). Another investigation found that α-MG reduced the synthesis of Aβ40 and Aβ42. α-MG did not affect the expression of enzymes implicated in the nonamyloidogenic and amyloidogenic pathways, but it considerably reduced the activities of β-secretase and likely γ-secretase, with IC_50_ values of 13.22 nmol/l and 16.98 nmol/l, respectively, in primary rat cerebral cortical neurons (44).

In primary cultured rat cortical cells, mangosteen pericarp water extract (<10 μg/ml) prevented neurotoxicity and the generation of ROS caused by Aβ (25-35) or excitatory amino acids. Mangosteen pericarp water extract suppressed caspase-3 activation and DNA fragmentation in cells treated with Aβ (25-35) or N-methyl-D-aspartate, indicating an anti-apoptotic effect. Mangosteen pericarp water extract also decreased lipid peroxidation and scavenged 1,1-diphenyl-2-picrylhydrazyl radicals, indicating that it is an anti-oxidant. Mangosteen pericarp water also inhibited the activities of β-secretase and AChE. The Morris water maze test was used to assess the effect of mangosteen pericarp water extract on memory impairment in scopolamine-treated mice. Mangosteen pericarp water extract administration (50, 100, or 300 mg/kg, 4 days, PO) considerably reduced the latency time to discover the platform and increased swimming time in the target quadrant (45). 

As a result, α-MG could be used as a lead compound for prevention or decreasing the severity of Alzheimer’s disease by mechanisms such as binding to Aβ and stabilizing the α-helical conformation, dissociating Aβ-(1-40) and Aβ-(1-42) oligomers, disrupting pre-formed fibrils while blocking fibril formation, preventing the increased intracellular ROS level, inhibiting the increased caspase-3 activity, and reducing the activities of β-secretase and likely γ-secretase.

***3-Nitropropionic acid***

Fungi (*Aspergillus flavus; Astragalus, Arthrinium*) and plants (*Indigofera endecapylla*) produce 3-nitropropionic acid, which is a natural poison. The succinate dehydrogenase (SDH; complex II) in the electron transportation chain, located inside the inner face of the mitochondrial membrane, is irreversibly inhibited by 3-nitropropionic acid, which causes neurotoxicity (46). Intoxication with 3-nitropropionic acid has no recognized antidote (47).

A study looked at the capacity of α-MG to scavenge ROS and its possible protective impact against the mitochondrial toxin 3-nitropropionic acid in primary cultures of cerebellar granule neurons. Singlet oxygen, superoxide anion, and peroxynitrite anion were all shown to be scavenged by α-MG in a concentration-dependent manner. α-MG, on the other hand, could not scavenge hydroxyl radicals or hydrogen peroxide. α-MG was also able to reduce the neuronal mortality caused by 3-nitropropionic acid in a concentration-dependent manner. The reduction of 3-nitropropionic acid-induced ROS production was linked to this protective effect (48).

Consequently, α-MG decreased the generation of ROS caused by mitochondrial toxin 3-nitropropionic acid *in vitro*. Therefore, more research is needed to see if α-MG can penetrate the blood-brain barrier and acquire adequate bioavailability in the brain to trigger a protective response against neurodegenerative diseases such as Alzheimer’s disease and Parkinson’s disease.

***Mangosteen and its compounds against chemical agents induced toxicity***

This section discusses the antidotal and protective actions of mangosteen and its compounds against a variety of chemical agents that cause toxicity, besides their significant protective mechanisms. 

***Anti-cancer drugs***

Besides mangosteen antidotal effects against chemotherapy agents-induced toxicity, it is interesting to mention that mangosteen phytochemicals (extracts, α-MG, β-MG, mangaxanthone B, and mangaphenone) have been shown in several *in vitro* and *in vivo* studies to suppress the growth and spread of cancer cells and to have an anti-proliferative and apoptosis-inducing impact on several human cancers, including breast, lung, liver, colon, oral, skin, leukemia, head and neck, prostate, and cervical cancers (49, 50).

***Doxorubicin***

Doxorubicin, one of the first two anthracyclines identified in *Streptomyces peucetius*, was isolated for the first time in the early 1960s. This drug is often used in chemotherapy to treat a variety of cancers such as carcinomas, sarcomas, hematological cancers, as well as solid tumors in children (20, 51). Despite its efficiency, it has a wide range of harmful side effects, the majority of which are a result of its intrinsic pro-oxidant activity. Doxorubicin is toxic to normal cells, including brain tissue (52).

The protective effect of a xanthone derivative of *G. mangostana* xanthones (200 mg/kg, single dose, IP) against doxorubicin-induced neuronal toxicity in male B6C3 mice was investigated and the findings revealed that xanthone could prevent doxorubicin from generating an increase in TNF-α, inducible nitric oxide synthase (iNOS) protein levels, and NO production in mononuclear cells. It also inhibited doxorubicin-mediated changes in pro- and anti-apoptotic proteins, reduced caspase-3 activity, and terminal deoxynucleotidyl transferase dUTP nick end labeling (TUNEL)-positive apoptotic cells, and reduced doxorubicin-mediated increases in protein carbonyl, 3-nitrotyrosine, and protein-bound 4-hydroxy-2´-nonenal (4-HNE) in brain tissues (53).

In conclusion, xanthone derivatives derived from mangosteen may be useful in avoiding tissue harm caused by ROS-producing chemotherapy medicines.

***Bleomycin***

Bleomycin is the generic name for a class of antibiotics isolated from the *Streptococcus verticillus* bacteria (54). Bleomycin is an important aspect of the treatment for a variety of tumors that can be cured, but it has a major drawback: lung damage like pulmonary fibrosis (55).

An *in vivo* study showed that α-MG administration (10 mg/kg/day, 14 days, i.g.) to male C57/BL6 mice considerably reduced bleomycin-induced extracellular matrix deposition in lung tissues. Furthermore, α-MG has been shown to reduce α-smooth muscle actin (α-SMA) and collagen I protein expression as well as its mRNA levels. In addition, α-MG suppressed the TGFβ1/Smad2/3 pathway and affected matrix metalloproteinase-9 and tissue inhibitor of metalloproteinase-1 (TIMP-1) protein expression in lung tissues. *In vitro* data showed that α-MG (1-50 nM, for 48 hr) enhanced phosphorylated-adenosine 5′monophosphate-activated protein kinase (p-AMPK)/AMPK but decreased the protein expression levels of α-SMA and collagen I as well as nicotinamide adenine dinucleotide phosphate oxidase-4 in activated primary lung fibroblasts (55). 

**Figure 5:**
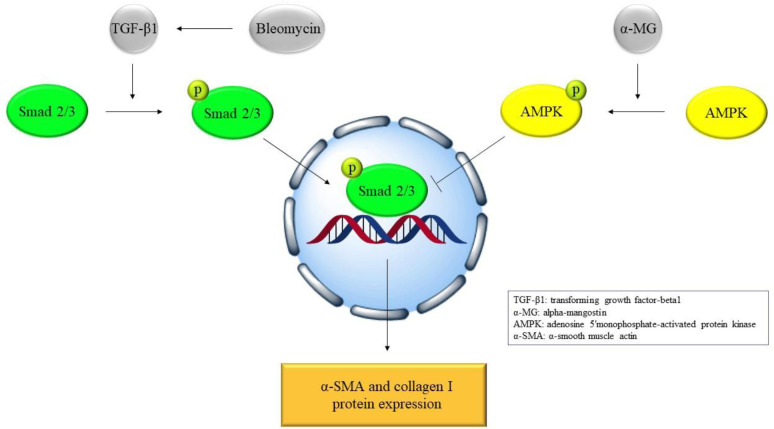
α-Mangostin antidotal molecular mechanism against bleomycin-induced pulmonary fibrosis

Overall, α-MG therapy reduced collagen formation, altered the redox status of lung fibroblasts, and alleviated bleomycin-induced pulmonary fibrosis in mice by targeting AMPK signaling (Figure 5).

***Cisplatin***

Cisplatin is a chemotherapy drug that is used to treat a variety of solid tumors, including testicular, ovarian, head, neck, colorectal, bladder, and lung cancers (56). Nephrotoxicity is the most common dose-limiting adverse effect of cisplatin (57).

α-MG (5 µM, for 24 hr) protects rats against cisplatin-induced kidney injury by diminishing the increase in ROS level, apoptotic cell death, GSH depletion, and increased p53 expression in Lilly laboratory culture porcine kidney (LLC-PK1) cells (58).

In another study, α-MG (2.5 μg/ml, for 48 hr) was found to protect HCT 116 human colorectal cancer cells from cisplatin-induced cytotoxicity due to the inhibition of ROS generation (59). Hence, this compound may have a cytoprotective impact against oxidative stress, irradiation, and chemical carcinogens, which could help prevent diseases like cancer.

In a human embryonic kidney (HEK293) cell model, the protective effect of α-MG against cisplatin-induced cytotoxicity was studied. It was discovered that α-MG (5, 10, 20, and 40 μM, for 24 hr) reduced cisplatin-induced cell death by lowering MDA levels and increasing GSH content. Following cisplatin encounter, α-MG dramatically reduced ROS overproduction, restored phosphoinositide 3-kinases (PI3K)/ protein kinase B (Akt) activation, and downregulated the c-Jun NH2-terminal kinase (JNK) pathways. Following that, α-MG significantly prevented the cleavage of caspases and poly-ADP-ribose polymerase, implicating ROS-mediated apoptotic pathways generated by cisplatin (60).

Cisplatin induces nephrotoxicity, which is complex and appears to be connected to free radical-induced damage (oxidative/nitrosative stress), inflammatory responses, fibrotic pathways, and a reduction in catalase activity. The renoprotective effect of α-MG on cisplatin-induced nephrotoxicity in male Wistar rats was investigated in another investigation. α-MG (12.5 mg/kg/day, i.g., for 10 days) reduced renal dysfunction, structural damage, oxidative/nitrosative stress, catalase expression, and TNF-α and TGFβ mRNA levels (53).

The protective effects of α-MG on cisplatin-induced damage in proximal tubule LLC-PK1 cells were investigated, and it was discovered that α-MG co-incubation (4 and 5 µM) inhibited cisplatin-induced cell death. Furthermore, α-MG reduced cisplatin-induced reductions in cell respiratory states,  the maximum capacity of the electron transfer system (E), and the respiration associated with oxidative phosphorylation. Cisplatin also reduced the protein levels of voltage-dependent anion channels and mitochondrial complex subunits, mitochondrial morphology changes, and mitochondrial mass (61).

In conclusion, α-MG attenuates cisplatin-induced nephrotoxicity or cytotoxicity by mechanisms like decreasing ROS level, increasing GSH content, and reducing the protein levels of mitochondrial complex subunits, mitochondrial morphology changes, and mitochondrial mass. Therefore, α-MG could be used as a protective agent against cisplatin toxicity.

***Streptozotocin***

Streptozotocin, a monofunctional nitrosourea derivative isolated from *Streptomyces achromogenes* in 1960, has broad-spectrum antibacterial action and antineoplastic characteristics, although its diabetogenic activities were not found until 1963. Through its damaging effects on pancreatic cells, it is commonly used to cause diabetes mellitus in experimental animals and due to its selective toxicity, it is also utilized to treat β-cell pancreatic tumors (62, 63).

The hypoglycemic activity of *G. mangostana *pericarp ethanolic extract in normoglycemic and streptozotocin-induced diabetic rats was evaluated in a study. Mangosteen pericarp ethanolic extract administration (50, 100, and 200 mg/kg, single-dose study (1 day) and multiple-dose study (29 days), PO) to male normoglycemic and streptozotocin-induced diabetic Sprague-Dawley rats remarkably reduced the blood glucose level. Furthermore, in the multiple-dose research, mangosteen pericarp ethanolic extract considerably increased the body weight of the rats when compared to the diabetic control group. Triglycerides, total cholesterol, LDL, very-low-density lipoprotein (VLDL), serum glutamic oxaloacetic transaminase (SGOT), serum glutamic pyruvic transaminase (SGPT), urea, and creatinine were all significantly reduced by this extract, while high-density lipoprotein (HDL) and total protein were significantly increased. In diabetic rats, there was a slight increase in the population of β-cells (26).

The anti-glycemic and anti-hepatotoxic benefits of mangosteen vinegar rind on a high-fat diet (HFD)/single dose STZ induced male institute of cancer research (ICR) diabetic mice are the topic of another investigation. When compared to the untreated diabetic control group, mangosteen vinegar rind administration (100 and 200 mg/kg, 1 week, PO) improved the levels of glucose, hepatic glycogen, lipid profile (lower total cholesterol, triglycerides, LDL levels, and higher HDL levels), oxidative stress (MDA level), anti-oxidant enzyme activity (SOD and CAT), and liver function biomarkers (ALT and AST) in HFD/streptozotocin-induced type II diabetic mouse models (64).

The anti-apoptotic and reno-protective properties of mangosteen vinegar rind aqueous extract against HFD/streptozotocin-induced type II diabetes nephropathy in ICR mice were investigated, and the findings revealed that acute mangosteen vinegar rind therapy (100 and 200 mg/kg, 1 week, PO) has a reno-protective effect on type II diabetes via reducing oxidative stress and apoptosis. This protective effect could be related to improvements in glucose level and lipid metabolism, mitochondrial integrity, oxidative stress reduction, inhibition of lipid peroxidation and inflammation, insulin sensitivity enhancement, and modulation of numerous apoptotic pathways (65).

Another study assessed the renal protective effects of γ-MG in male streptozotocin-induced diabetic BALB/c mice, and the results showed that γ-MG administration (1, 2, and 4 mg/kg, 2 weeks) was able to significantly lower plasma blood urea nitrogen (BUN) and creatinine, as well as ameliorate diabetic mice’s impaired renal proximal tubular cells (66).

Therefore, the constituents of *G. mangostana *could be a potential candidate for the management of hyperglycemia, hyperlipidemia, and diabetic nephropathy through several mechanisms like improvement in the level of glucose, hepatic glycogen, lipid profile (lower total cholesterol, triglycerides, LDL levels, and higher HDL level), oxidative stress (MDA level), anti-oxidant enzyme activity (SOD and CAT), liver function biomarkers (ALT and AST) and mitochondrial integrity, inhibition of lipid peroxidation and inflammation, insulin sensitivity enhancement, and modulation of numerous apoptotic pathways and lowering plasma BUN and creatinine.

***Antibiotics***

*Isoniazid*

Isoniazid (isonicotinic acid hydrazide) is one of the main therapies for tuberculosis, which is caused by infection with *Mycobacterium tuberculosis*. Isoniazid is not destructive to the bacterial cell itself, but it is a prodrug that is activated by the mycobacterial catalase-peroxidase enzyme (67). Hepatotoxicity and a potentially deadly liver injury are linked to isoniazid usage. Hepatotoxicity is frequently accompanied by nausea and right upper quadrant stomach discomfort, but it can also be asymptomatic, and the diagnosis is based on bilirubin and SGPT levels in the serum (68). 

The impact of the ethanol extract of *G. mangostana* peel on isoniazid-induced liver damage in rats was examined in the research. The findings of administering mangosteen peel ethanol extract (250 and 500 mg/kg/day, 35 days) to male Wistar rats revealed that 500 mg/kg of ethanol extract of *G. mangostana* peel reduced isoniazid-induced liver damage in rats by lowering TGF-β1, SGPT level, and liver fibrosis (69).

As a result, ethanol extracts of *G. mangostana* peel prevent isoniazid-induced liver damage in rats by lowering TGF-β1, SGPT levels, and liver fibrosis.

***Analgesics***

*Acetaminophen*

Acetaminophen is one of the most widely used and well-tolerated pain relievers in the world. Its exact mechanism of action is unknown, however, it appears to selectively inhibit cyclooxygenase (COX) in the brain, and its capacity to relieve fever and discomfort is a result of this effect. In the central nervous system, it may also suppress PGs production. Acetaminophen has an antipyretic effect by acting directly on the hypothalamus (70). Because of its widespread availability, acetaminophen is commonly linked to overdoses, both deliberate and unintentional, resulting in severe liver damage and even acute liver failure (71).

α-MG treatment (100, 200 mg/kg/day, 7 days, i.g.) reduces the adverse effects of overdose acetaminophen-induced hepatic injury in male ICR mice, partly by restoring anti-oxidative activity and modulating inflammation, apoptosis, and autophagy via increasing serum aminotransferase levels and GSH content, reducing MDA, inhibiting increases in TNF-α and IL-1β, and the protein expression of autophagy-related microtubule-associated protein light chain 3 and BCL2/adenovirus E1B protein-interacting protein 3. Furthermore, the protective effect of α-MG on acetaminophen-induced acute liver injury might be attributed to changes in the liver Akt/mTOR signaling pathway (72).

Moreover, α-MG protective effect against acetaminophen-induced acute liver injury was evaluated in another study in male ICR mice. α-MG (12.5 and 25 mg/kg/day, 6 days, i.g.) reduced serum levels of ALT, AST, TNF-α, IL-1β, IL-6, and hepatic MDA, while restoring hepatic GSH, SOD, and CAT activity. Moreover, α-MG pretreatment effectively decreased acetaminophen-induced phosphorylation of ERK, JNK, and p38 MAPK, which was associated with alterations in TNF-α, IL-1β, and IL-6 levels; phosphorylation of IκBα and translocation of NF-κBp65 were also suppressed by α-MG (38).

In another study, tovophyllin A (50 and 100 mg/kg/day, 5 days) protected male BALB/c mice against acetaminophen-induced hepatic damage through its significant anti-oxidant (reversing the elevation of MDA and 4-hydroxynonenal levels, recovering hepatic GSH, SOD, and CAT, and decreasing the level of NOx) and anti-inflammatory effects, which increased Nrf2 activation and disrupted the NF-κB pathway (73).

Consequently, α-MG and tovophyllin A reduce acetaminophen-induced liver damage by mechanisms like lowering serum levels of ALT, AST, TNF-α, IL-1β, IL-6, and hepatic MDA, boosting Nrf2 activation, and disrupting the NF-κB pathway. As a result, α-MG and tovophyllin A may be used as an antidote to acetaminophen toxicity.

***β*** ***-*** ***Adrenergic*** ***agonist***

*Isoproterenol*

Isoproterenol is a nonselective sympathomimetic β-adrenergic agonist manufactured synthetically. It is most commonly used to treat bradycardia, thioridazine-induced torsade de pointes, and heart block. Isoproterenol produces highly cytotoxic free radicals through auto-oxidation, which causes oxidative stress, resulting in gradual mitochondrial damage and changes in cardiac biochemical parameters, leading to heart injury (74, 75). 

Isoproterenol induction resulted in a considerable rise in the activity of serum and cardiac lysosomal hydrolases (β-D-glucuronidase, β-D-galactosidase, β-D-N-acetylglucosaminidase, acid phosphatase, and cathepsin-D) in adult male Wistar rats. In the hearts of isoproterenol-administered rats, the aberrant activity of membrane-bound phosphatases (Na^+^-K^+^ ATPase, Ca^2+^ ATPase, and Mg^2+^ ATPase) was noted, as well as a considerable rise in cardiac sodium and calcium levels with a decrease in potassium levels. TNF-α and COX-2 expression in the heart of isoproterenol-intoxicated rats were considerably higher. When compared to the isoproterenol-intoxicated group of rats, pre-co-treatment with α-MG (200 mg/kg, 8 days, PO) considerably reduced these anomalies and restored levels to near normalcy (76).

The induction of adult male Wistar rats with isoproterenol caused a considerable increase in lipid peroxidation, serum marker enzymes (lactate dehydrogenase (LDH), creatine phosphokinase (CPK), glutamate oxaloacetate transaminase (GOT), and glutamate pyruvate transaminase (GPT)), and a significant decrease in endogenous anti-oxidant activity (SOD, CAT, glutathione peroxidase (GPx), glutathione-S-transferase (GST), and GSH). When compared to the individual treatment groups, pre-treatment with α-MG (200 mg/kg/day, 6 days, PO) before isoproterenol administration and 2 days after ISO administration considerably reduced these alterations (77).

The potential role of mangosteen in isoproterenol-induced myocardial infarction in adult male albino rats was investigated. The administration of mangosteen (18 mg/200 mg, p.o.) resulted in a partial improvement in heart muscle fibers as well as a reduction in inflammatory cellular infiltration (78).

As a result of these findings, mangosteen and α-MG can be used as a cardiotoxic preventive against β-adrenergic catecholamine-induced myocardial toxicity and oxidative stress.

***Anticholinergic***

*Scopolamine*

Scopolamine is a muscarinic receptor blocker that impairs cholinergic neurotransmission, causing memory loss in Alzheimer’s disease patients (79).

γ-MG (3~10 μM, for 24 hr) protected rat cerebrocortical cells from H_2_O_2_- or xanthine/xanthine oxidase-induced oxidative neuronal death and reduced the formation of ROS generated by these oxidative insults. It also prevented H_2_O_2_-induced DNA fragmentation and caspase-3 and 9 activations, confirming its antiapoptotic properties. Furthermore, γ-MG was discovered to efficiently prevent lipid peroxidation, the generation of 1,1-diphenyl- 2-picrylhydrazyl radicals, and the activity of β-secretase. The effect of γ- MG on scopolamine-induced memory impairment in ICR mice was assessed using the passive avoidance test, and it was discovered that γ-MG (10 and 30 mg/kg, PO) significantly reduced scopolamine-induced memory impairment (80).

Mangosteen extract (200 g/ml, for 3 hr) might partially counteract the effects of H_2_O_2_ on cell survival, ROS level, and caspase-3 activity in SK-N-SH cell cultures. Mangosteen extract (200, 400, or 800 g/ml, for 3 hr) lowered SK-N-SH cells’ AChE activity to around 60% of the control. The Morris water maze and passive avoidance tests were utilized to examine the memory of male ICR mice in an *in vivo* investigation. Mangosteen extract (100 mg/kg/day, PO) for the passive avoidance test and the Morris water maze test improved the animal’s memory and antagonized the effect of scopolamine on memory. The mangosteen extract therapy counteracted the rise in ROS level and caspase-3 activity in the brains of scopolamine-treated mice (81).

Therefore, γ-MG and mangosteen extract might be used as a preventive treatment against scopolamine-induced memory impairment through mechanisms such as reduction in ROS production and prevention of caspase-3 and 9 activations.

***Thioacetamide***

Thioacetamide contains thiono-sulfur and has been employed as a fungicide, an organic solvent, a rubber accelerator, and a motor oil stabilizer (82). Thioacetamide has been shown to cause liver fibrosis and cirrhosis in experimental animals (15).

In male Wistar rats, the effects of α-MG on thioacetamide-induced liver cirrhosis were examined. α-MG (100 mg/kg, 3 times per week, 4 weeks, IP) reduced fibrotic nodules and lowered AST and ALT levels in the blood. It also decreased the risk of liver fibrosis by lowering p53 expression (83).

Thioacetamide caused histologically detectable liver damage and fibrosis in rats in another investigation. It also elevated TGF-β1, α-SMA, and TIMP-1 immunohistochemically detectable levels. The effects of thioacetamide treatment alone were avoided or ameliorated by co-administration of α-MG (5 mg/kg, 3 times per week, 4 weeks, IP) with thioacetamide (84).

Consequently, α-MG acts as a protective agent against thioacetamide-induced liver fibrosis and cirrhosis by reducing fibrotic nodules, lowering AST and ALT levels, lowering p53 expression, and ameliorating the elevated TGF-β1, α-SMA, and TIMP-1.

***Lead***

Lead is found all over nature. It may be found in a variety of forms, including bullets, inorganic compounds like lead oxide, lead chromate, and lead sulfide, and organic compounds like tetraethyl lead (85). Abdominal discomfort, constipation, anemia, hearing loss, exhaustion, neuropathy and neurotoxicity, encephalopathy, renal disorders, abortions, osteopenia, and even mortality are among the symptoms of lead poisoning (86).

Xanthone (100 and 200 mg/kg, 38 days, PO) alleviated lead-induced neurotoxicity in ICR mice, in part by suppressing oxidative damage and reversing AChE activity. Forced swimming and Morris water maze tests have shown that it has substantial protective benefits against lead-induced learning deficits and memory loss (87). 

The potential preventive benefits of xanthones against lead acetate-induced chronic renal disease in ICR mice were investigated. Xanthones (100 and 200 mg/kg/day, 38 days, PO) scavenged the radicals 2,2-diphenyl-1-picrylhydrazyl, superoxide, hydroxyl, and NO. Lead acetate-induced oxidative stress, renal dysfunction, inflammatory markers (plasma TNF-α concentration and protein expression of TNF-α, COX-2, and iNOS in kidney tissue), and kidney apoptosis were reduced by co-treatment with xanthones. The tissue architecture was significantly enhanced as a result of the therapy. An *in-silico* prediction of activity investigation revealed that xanthones’ protective effects may be owing to their ability to activate Nrf2, control intracellular [Ca^2+^], and downregulate the NF-κB, MAPK pathway (88).  

As a result, by reducing oxidative damage, reversing AChE activity, and lowering inflammatory markers, xanthones protect against lead and lead acetate-induced toxicity.

The protective effects of mangosteen and its main constituents against some other chemical toxicants can be found in Table 2.

***Mangosteen’s toxicity***

In previous research, the lethal dose (LD_50_) for intraperitoneal administration of the crude methanolic extract to female BALB/c mice was determined to be 1000 mg/kg, while the appropriate dose for short-term studies should be 200 ≤ mg/kg (27). Rahmayanti *et al*. determined that the LD_50_ of the ethyl acetate fraction from mangosteen pericarp extract was >15.480 mg/kg after oral administration to female Sprague-Dawley rats (89). Another study found that oral administration of mangosteen skin extract to BALB/c mice in doses of up to 5000 mg/kg was not toxic and could be used as a natural herbal medicine (90). It has also been reported that intraperitoneal administration of α-MG and mangosteen extract to mice resulted in LC_50_ of 150 and 231 mg/kg, respectively (91). The acute and subchronic toxicity of a tannin-rich extract from the mangosteen fruit pericarp was studied in Swiss albino mice via intragastric administration, and the results showed that the extract at the doses tested (2 and 5 g/kg for acute toxicity and 400, 600, and 1,200 mg extract/kg for subchronic toxicity) had no significant negative effects in the experimental animals (92).

**Table 1:** Protective effects of mangosteen and its main constituents against LPS-induced nervous system inflammation and murine macrophage inflammation

Type of inflammation	Model	Protective agent	Results	Reference
**Nervous system inflammation**	young female C57BL/6J mice	α-mangostin (40 mg/kg/day, 14 days, p.o.)	↓ the levels of pro-inflammatory cytokine IL-6, COX-2, and translocator protein in the brain	(93)
**Nervous system inflammation**	BV-2 cell line	α-mangostin (100-500 nM, for 1h)	↓ LPS-induced pro-inflammatory cytokine production and iNOS expression ↓ microglia migration and phagocytosis in response to LPS, LPS-induced microglia-mediated neuronal dendritic damage, TLR4 expression, as well as TAK1 and NF-𝜅B activation	(94)
**Nervous system inflammation**	male C57BL/6 mouse	α-mangostin (50 mg/kg/day, 14 days, p.o.)	↓ LPS-induced microglial activation and neuroinflammation, as well as LPS-induced activation of the TLR4/TAK1/NF-𝜅B signaling pathway, LPS-induced dendritic damage, LPS-caused learning and memory impairments	(94)
**murine macrophage inflammation**	RAW264.7 cells	*G. mangostana* peel extract (5, 10 and 20 μg/mL) and its compounds ( α-mangostin and γ-mangostin) (25, 50 and 75 μM)	↓ COX-2, IL-6, IL-1, and NO production	(95)

IL-6: Interleukin-6; COX-2: Cyclooxygenase-2; iNOS: Inducible nitric oxide synthase; LPS: Lipopolysaccharide; TLR4: Toll-like receptor 4; TAK1: Transforming growth factor-activated kinase 1; NF-𝜅B: Nuclear factor-𝜅B; IL-1: Interleukin-1; NO: Nitric oxide

**Table 2:** Protective effects of mangosteen and its main constituents against chemical toxicants

Compound	Study model	Protective agent	Results	Reference
**Rotenone**	*In vivo,* Sprague Dawley rats	α-mangostin (10 mg/kg, 21 days, i.p)	↑ antioxidant enzyme levels ↓ phosphorylated α-synuclein levels, TH+- dopaminergic neuronal loss in the substantia nigra pars compacta, memory impairments	(31)
**Paraquat and NaNO**_2_	*In vitro*, Chinese hamster lung cells	Xanthone (0, 0.1, 0.5, 1, 5, 10 µM, 30 min prior to paraquat and NaNO_2_ exposure)	↓ sister chromatid exchange and decreased cell cycle rate	(96)
**Iodoacetate**	*In vitro*, cerebellar granule neurons	α-mangostin (8, 12, and 14 μM, for 16 hours)	↑ antioxidant induction of heme oxygenase-1 ↓ ROS generation	(97)
**3.8. tert-Butyl hydroperoxide**	*In vitro*, human normal hepatocytes (HL-7702)	γ-mangostin (0.63, 1.25, 2.50, and 5.00 μM)	↑ SOD amount ↓lipid peroxidation, GSH levels, loss of mitochondrial membrane potential	(98)
**Cigarette smoke**	*In vivo, *rats	mangosteen peel extract (150, 300, and 600 mg/kg, 21 days, p.o.)	↑ SOD activity ↓ MDA level	(99)
**Iodixanol**	*In vitro, *LLC-PK1 cells	α-mangostin (2.5 and 5 μM, 3 h)	↑cell viability ↓ phosphorylation of p38, ERK, and caspase-3 cleavage	(100)

ERK: extracellular-signal-regulated kinase; GSH: glutathione; MDA: malondialdehyde; ROS: reactive oxygen species; SOD: superoxide dismutase

## Conclusion

In this review, we summarized the findings from several *in vitro* and *in vivo* studies on *G. mangostana *and its principal components, particularly α-MG, γ-MG, and tovophyllin A, to highlight mangosteen’s antidotal and protective activities against biological and chemical toxicities. Mangosteen protects against a variety of biological toxins, including LPS, ovalbumin, β-amyloid, and 3-nitropropionic acid, through different mechanisms like anti-inflammatory and anti-apoptotic properties, histological condition improvement, and reduction of oxidative stress. It also has a protective role against chemical toxic agents including anti-cancer drugs, β-adrenergic agonists, anticholinergic, isoniazid, acetaminophen, thioacetamide, and lead. Mangosteen and its constituents exert their effects primarily through different mechanisms such as anti-oxidant, radical scavenging, anti-apoptotic properties, anti-inflammatory effects, and the regulation of the renal, hepatic, and cardiac enzymes. All current evidence shows that *G. mangostana* and its components have extremely promising benefits and that more study, including clinical trials, is required.

## Authors’ Contributions

HH Study conception, design and supervision of the research; MGR Critical revision of the paper, supervision of the research; AMZ Preparation of the original draft. All authors have agreed to the contents and approved the final version for publication

## Data availability statement

The data that support the findings of this study are available from the corresponding author upon responsible request.

## Conflicts of Interest

The authors declare no conflicts of interest. 
